# Cerebellum in Alzheimer's disease and other neurodegenerative diseases: an emerging research frontier

**DOI:** 10.1002/mco2.638

**Published:** 2024-07-13

**Authors:** Cui Yang, Guangdong Liu, Xi Chen, Weidong Le

**Affiliations:** ^1^ Institute of Neurology Sichuan Provincial People's Hospital School of Medicine University of Electronic Science and Technology of China Chengdu China

**Keywords:** Alzheimer's disease, cerebellum, dementia, neurodegenerative diseases, pathological and biochemical profiles, structural and functional neuroimaging

## Abstract

The cerebellum is crucial for both motor and nonmotor functions. Alzheimer's disease (AD), alongside other dementias such as vascular dementia (VaD), Lewy body dementia (DLB), and frontotemporal dementia (FTD), as well as other neurodegenerative diseases (NDs) like Parkinson's disease (PD), amyotrophic lateral sclerosis (ALS), Huntington's disease (HD), and spinocerebellar ataxias (SCA), are characterized by specific and non‐specific neurodegenerations in central nervous system. Previously, the cerebellum's significance in these conditions was underestimated. However, advancing research has elevated its profile as a critical node in disease pathology. We comprehensively review the existing evidence to elucidate the relationship between cerebellum and the aforementioned diseases. Our findings reveal a growing body of research unequivocally establishing a link between the cerebellum and AD, other forms of dementia, and other NDs, supported by clinical evidence, pathological and biochemical profiles, structural and functional neuroimaging data, and electrophysiological findings. By contrasting cerebellar observations with those from the cerebral cortex and hippocampus, we highlight the cerebellum's distinct role in the disease processes. Furthermore, we also explore the emerging therapeutic potential of targeting cerebellum for the treatment of these diseases. This review underscores the importance of the cerebellum in these diseases, offering new insights into the disease mechanisms and novel therapeutic strategies.

## Graphical abstract

The cerebellum has a highly organized laminar flow structure and tightly anatomical circuits connecting with the cerebral cortex and subcortical regions, through which it is involved in the regulation of movement, balance, and cognition. To understand the role of the cerebellum in Alzheimer's disease (AD), we propose a comprehensive review highlighting a succinct synopsis of the clinical manifestation, neuropathology, neurophysiology, neuroimaging, and electrophysiology features associated with cerebellar dysfunction in AD, thereby underscoring the cerebellum as an emerging frontier for AD research. *Abbreviations*: Aβ, amyloid‐β; EEG, electroencephalography.

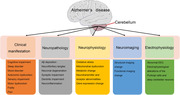

## 1 INTRODUCTION

The cerebellum constitutes a significant component of the brain,^1^ characterized by a highly organized laminar structure comprising three cortical layers and one white matter layer, each with distinct cell distributions.^2^, ^3^ Historically, spanning nearly two centuries, the cerebellum has predominantly been associated solely with motor and balance functions,^4^ with nonmotor functions often overlooked. However, exciting and shocking discoveries have reshaped perspectives, revealing the cerebellum's involvement in nonmotor functions.^5^ As early as 1776, Vincenzo Malacarne speculated on the relationship between intelligence and the number of layers in cerebellum,^4^ followed by Giuseppe Moruzzi's proposition in 1986 regarding the cerebellum's role in higher mental functions.^6^ The report on the cerebellar cognitive affective syndrome in 1998 further catalyzed the research into the cerebellum's cognitive perspectives.^7^ Advancements in biotechnology have broadened the current insights into cerebellum, particularly its nonmotor functions including cognitive impairment, sleep disorders, mood disorders, autonomic dysfunction, and sensory impairment, stimulating interest in its functional diversity.^8^, ^9^, ^10^

Alzheimer's disease (AD), along with the various forms of dementia including vascular dementia (VaD), Lewy body dementia (DLB), and frontotemporal dementia (FTD), as well as other neurodegenerative diseases (NDs) such as Parkinson's disease (PD), amyotrophic lateral sclerosis (ALS), Huntington's disease (HD), and spinocerebellar ataxias (SCA), are debilitating conditions characterized by progressive dysfunction or structural alteration within the associated neuronal systems.^11^, ^12^, ^13^ Traditionally, research on those diseases has primarily focused on the cerebrum or spinal cord, not the cerebellum. However, recent research surrounding the underlying pathological mechanisms and treatment strategies in the above diseases has discovered the importance of cerebellum in the pathogenesis of AD and otherNDs. As our understanding of cerebellar function deepens, it becomes apparent that the cerebellum may influence some clinical presentations of these diseases, encompassing both motor dysfunction and nonmotor dysfunctions, including cognitive impairment, sleep disorders, mood disorders, autonomic dysfunction, and sensory impairment. Consequently, there is a growing interest in exploring whether the cerebellum plays a role in the progression of those diseases, leading to a surge in research activities. The emerging body of research has started to unveil the connections between cerebellum and these diseases. Given the elusive nature of their pathogenesis and limited treatment options, further exploration of the cerebellum's involvement could provide valuable insights into the disease mechanisms and may identify new therapeutic targets.

This review aims to comprehensively examine existing literatures to elucidate the relationship between cerebellum and NDs. Specifically, it systematically explores the clinical evidence, pathological and biochemical profiles, structural and functional neuroimaging data, and electrophysiological findings associated with cerebellum in AD, other dementias, and other NDs.

## 2 CEREBELLUM AND AD

AD is the main dementia in the world and a neurodegenerative condition prevalent in the elderly.^14^, ^15^ Despite the research progress since Alois Alzheimer's inaugural description of the disease in 1906,^16^ the consensus amongst researchers is that AD pathology influences mostly in the cortex and select subcortical brain regions, including the frontal, temporal, and parietal lobes, portions of the cingulate gyrus, the hippocampus, and certain brainstem nuclei.^17^

**FIGURE 1:**
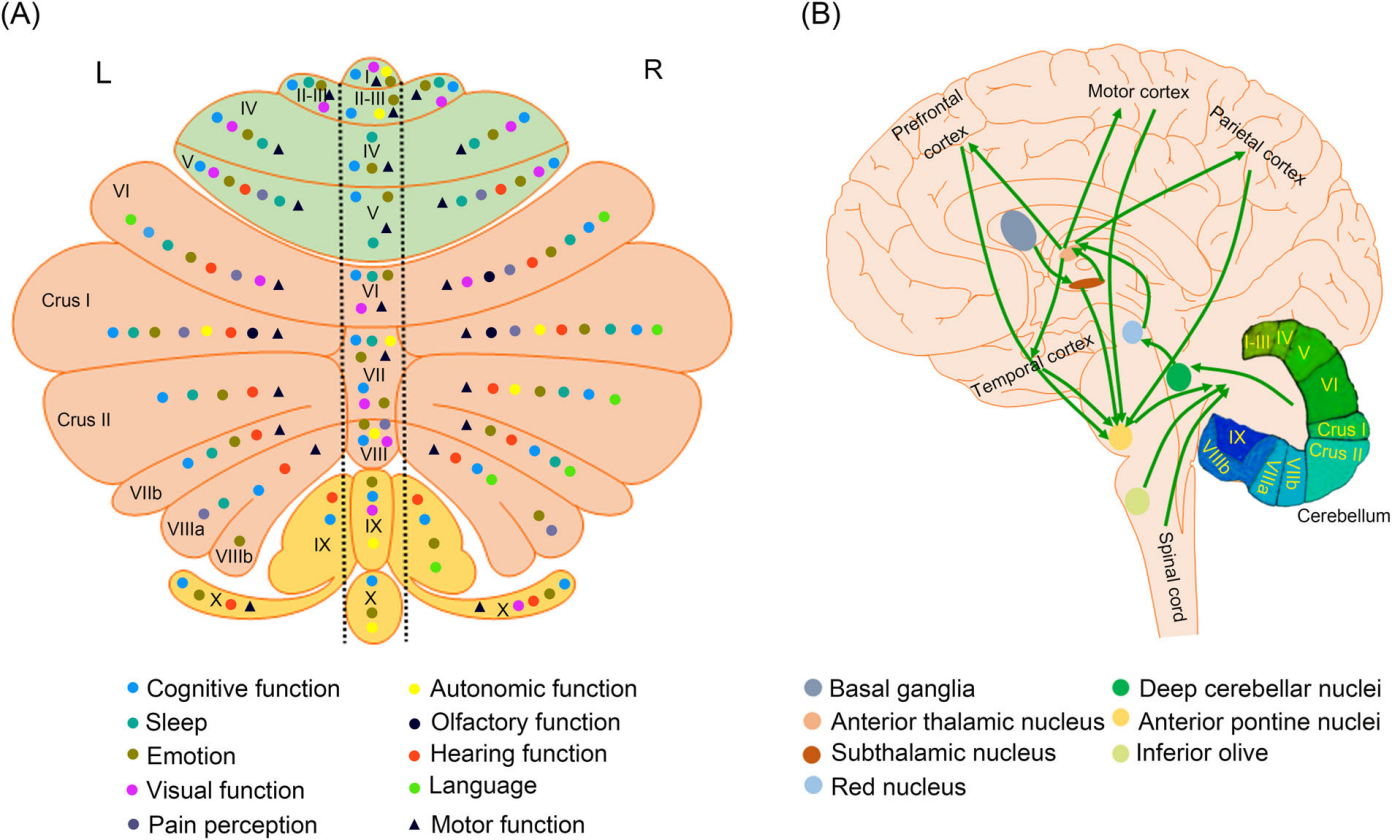
Diagram of functions and connectivity of the human cerebellum. (A) Functional distribution map of the human cerebellum. The figure above shows the functional regions of the cerebellum for cognitive function, sleep, emotion, visual function, pain perception, autonomic function, olfactory function, hearing function, language, and motor function. (B) The figure represents schematically the bidirectional connectivity between the cerebellum and the telencephalon, in particular with the cerebral cortex. Telencephalic projections from the cortex and basal ganglia (through the subthalamic nucleus) and limbic areas are relayed to the cerebellum through the anterior pontine nuclei. Pictorial representation of the anatomical reciprocal connections between the inferior olive, spinal cord, and cerebellum. The cerebellum in turn sends its output through the deep cerebellar nuclei, red nucleus, and anterior thalamic nucleus to various telencephalic areas including the motor cortex, the prefrontal cortex, the parietal cortex, and the temporal cortex. These connections, which are supported by anatomical and functional data, form several bidirectional cerebello‐thalamo‐cerebro‐cortical circuits.

However, growing evidence highlights the involvement of the cerebellum in AD. The cerebellum plays an important role in cognitive function, and damage to the cerebellum can lead to cognitive dysfunction as seen in patients with AD.^18^ Notably, the cerebellum shares extensive and bidirectional connections with a large portion of the area in the brain (Figure 1). That is, the lesion, in any part of the brain, could influence the cerebellum through various mechanisms.^19^ Further, it has been reported that the clinical symptoms of AD could be improved by stimulating cerebellum.^20^ Thus, the cerebellum seems to have garnered significant attention to become a new entry point. Certainly, a detailed understanding of the cerebellum status in AD can provide a basis for further research.

### 2.1 Clinical manifestations linking cerebellum to AD

AD is often characterized by cognitive impairment,^21^ which may be accompanied by other symptoms, including sleep disturbances,^22^ mood disorders,^23^ autonomic dysfunctions,^24^ specific sensory disorders (olfaction, hearing, vision),^25^ and others. Neurodegeneration in the cortex and hippocampus can contribute to the cognitive and noncognitive dysfunctions observed in AD. Numerous studies have shown that the cerebellum plays a crucial role in the generation and maintenance of the aforementioned cognitive and noncognitive functions. Furthermore, abnormalities in the structure and function of cerebellum also lead to impairments in cognitive and noncognitive functions.^8^, ^9^

#### 2.1.1 Cognitive impairment and cerebellum

Cognitive impairment, the core clinical symptom of AD, manifests as memory impairment, executive dysfunction, visual‐spatial disorder, language disorders, and psychological changes in personality, behavior, or demeanor. A range of studies have demonstrated that nearly the entire cerebellum is involved in cognitive function (Figure 1).^26^, ^27^, ^28^, ^29^, ^30^, ^31^, ^32^ Cerebellar activation is associated with various tasks, including those related to working memory, learning, attention, and executive control.^9^, ^33^ In particular, Crus I and Crus II contribute to parallel cortico‐cerebellar circuits that regulate executive control, salience detection, and scene memory/self‐reflection.^34^ Additional studies have indicated that the neocerebellum participates in the executive control network, which is associated with working memory.^34^, ^35^ Furthermore, different regions of the cerebellum may be involved in regulating various types of cognitive functions. The cerebellum plays a role in coordinating motor and nonmotor speech processes.^36^, ^37^, ^38^, ^39^ During nonmotor speech, lateral and posterior cerebellar regions are mainly involved.^38^ A study has documented overall higher cerebellar prediction accuracy compared with the whole brain, with distinct structural signatures of higher anterior cerebellar contribution to mild cognitive impairment (MCI) and higher posterior cerebellar contribution to mild/moderate stages of AD for each tissue property.^40^ Therefore, the cerebellum has an anatomical basis for regulating cognitive function, and cerebellar damage can lead to cognitive impairment.^41^

#### 2.1.2 Sleep disorders and cerebellum

Sleep is an important brain function that supports cognitive processes.^42^ Approximately 45% of AD patients experience varying degrees of sleep disorders.^43^ The cerebellum exhibits different patterns of sleep activity compared with the cerebral cortex, and these activities may influence sleep structure. Strong interconnections exist between the cerebellum and the cerebral cortex via the thalamus, pontine nuclei, mesencephalic junction, and inferior olive (Figure 1). The reverberating activity in this circuit is significantly influenced by sleep–wake states.^44^, ^45^, ^46^

The cerebellum plays a role in regulating sleep.^47^, ^48^, ^49^, ^50^, ^51^ Sleep disturbances can lead to cerebellar dysfunction and vice versa. Cerebellum volume is reduced in patients with primary sleep disorders, such as chronic insomnia, fatal familial insomnia, or obstructive sleep apnea with daytime sleepiness.^52^ Similarly, patients with rapid eye movement (REM) sleep behavior disorder, a condition characterized by motor activity during REM sleep, exhibit reduced volume in the anterior lobes of the cerebellar cortex and cerebellar nuclei.^53^ Patients with primary cerebellar dysfunction can present with multiple sleep disorders.^52^, ^54^ Sleep contributes to cerebellar learning and consolidation, which depends on temporal and procedural memory formation, as well as spatiotemporal prediction of motor actions.^55^, ^56^ In sleep‐dependent sequential learning tasks, such as sequential finger tapping and sequential reaction time tasks, sleep improves tapping speed by 10−20%. Subsequent postsleep cerebellar activity correlates with the consolidation of learned rhythmic motor activity.^57^ The increase in finger‐to‐thumb task performance is associated with the amplitude of sleep spindles and the depth of REM sleep,^58^ whereas the decrease in the duration of sleep spindles with age is not only associated with the decrease in cerebellar gray matter volume but also with the deficit in motor memory consolidation.^59^ While awake, the part of the cerebellum that is activated during a series of reactivity time tasks is more active during REM sleep in subjects who have previously been trained on the task than in subjects who have not been trained on the task.^60^, ^61^, ^62^ Sleep deprivation not only affects the acquisition of classical blink conditioning, but the acquisition of classical blink conditioning depends on the intact cerebellum.^63^ There is a functional connection (FC) between the lateral cerebellar nucleus and superior temporal sulcus of neocortex.^64^ Thus, in AD, the cerebellum may influence sleep structure.

#### 2.1.3 Mood disorders and cerebellum

Mood disorders are often present in AD.^65^ It is important to highlight that the prevalence of depression among AD patients can reach as high as 50%,^66^ while anxiety affects more than 44% of individuals with AD.^67^ The cerebellum is a complex and integral part of the nervous system that processes emotions.^68^ Further, the cerebellum is involved in both the early stages of perception and recognition of emotional cues and the later stages of integration of emotional assessment.^68^ Posterior vertical and paranormal cerebellum correlate with primary emotions.^69^ Further, negative emotions are associated with activity in the left VI, right IV/V, and bilateral Crus I. Positive emotions are associated with activity in the right VI.^32^, ^38^ Additionally, there is evidence showing that the cerebellum participates in tasks in which emotion is intertwined with cognition and reasoning.^70^ Cerebellar disorders are often accompanied by depressive symptoms.^71^, ^72^ Meanwhile, several studies have shown that patients with a range of mood disorders display changes in the thickness, density, or volume of the cerebellum.^71^, ^73^

#### 2.1.4 Autonomic dysfunction and cerebellum

Autonomic dysfunction is often present in AD.^74^ The cerebellum is involved in regulating the autonomic nervous system.^75^, ^76^, ^77^, ^78^, ^79^ However, the cerebellum does not work alone; instead, it is intricately interconnected and engaged with other components of the central nervous system.^80^, ^81^ Researchers Golanov and Reis^82^ have discovered a potential link between vasomotor changes and electroencephalographic bursts, suggesting that cerebellar influence on visceral functions may be partially mediated through the cerebral cortex.^82^ Additionally, there is a direct pathway from the hypothalamus to the cerebellar cortex and nuclei, indicating a potential connection in controlling visceral responses.^83^ It is worth noting that the cerebellum also maintains a sparse but direct association with the periaqueductal gray^84^ and the dorsal pons/pontine.^85^ Autonomic dysfunction could cause abnormal cerebellar function,^86^ and the abnormal cerebellum could result in autonomic dysfunction.^87^

#### 2.1.5 Sensory impairments and cerebellum

High‐level cortical impairments in olfactory, hearing, and vision may lead to sensory dysfunction due to early neuropathological changes in AD.^88^ Sensory dysfunction appears earlier than memory and cognitive decline and gradually worsens with the progression of AD.

Approximately 85−90% of AD patients exhibit olfactory dysfunction,^88^ with the cerebellum being implicated in its pathophysiology.^89^, ^90^, ^91^ Studies have revealed that odorants, such as vanillin and propionic acid, could induce substantial activation in the posterior lateral hemisphere of the cerebellum, and this activation is contingent upon concentration.^89^ Further studies have shown that odor intensity discrimination and odor quality discrimination acted on the left insula and right cerebellum.^90^ Olfactory discrimination is positively correlated with the lobular VI grey matter volume.^92^

AD is strongly associated with hearing loss, affecting around 81% of AD patients.^93^ The cerebellum has been identified as a critical regulator of auditory function,^94^, ^95^, ^96^ as it establishes connections with neighboring auditory structures and various central systems to modulate cochlear activity.^97^, ^98^ Hearing impairment is known to significantly affect cerebellar structure and function.^99^, ^100^ Conversely, cerebellar damage can lead to hearing impairment,^101^, ^102^ as exemplified by progressive bilateral sensorineural hearing loss following a cerebellar tumor diagnosis.^101^ Moreover, AD patients with hearing loss often display reduced cerebellar volumes bilaterally compared with those without hearing loss.^103^

The degenerative process of AD is often accompanied by early deficits in the visual system.^25^ The cerebellum is associated with various types of eye movements, encompassing both immediate adjustments and long‐term adaptation.^104^ Additionally, the cerebellum is also connected to the regions that process object vision.^105^ Meanwhile, studies have shown the localization of visual area within the cerebellum in several regions.^95^, ^96^, ^106^ Cerebellum relies heavily on target vision for both language processing and mathematics.^107^ Hence, cerebellar dysfunction is likely to contribute to the visual impairments observed in AD patients.

Furthermore, AD presents with a range of additional clinical manifestations, encompassing motor dysfunction, frailty, and pain.^108^, ^109^, ^110^ Research has implicated the cerebellum's contribution to motor dysfunction,^38^, ^111^, ^112^, ^113^, ^114^, ^115^, ^116^, ^117^, ^118^, ^119^, ^120^ frailty, and pain.^9^, ^121^

Consequently, the cerebellum seems to be integral to virtually most clinical signs and symptoms of AD (Table 1). However, there is no clear research report on whether the structural and functional changes of the cerebellum are inevitable synergistic or even protective with the clinical manifestations of AD. In addition, the relatively uniform anatomy and physiology of the cerebellar cortex have forced us to consider whether there is a distribution pattern between cerebellar partitioning and clinical manifestations of AD. Therefore, we map the localization of nonmotor and motor functional areas within the cerebellum (Figure 1). We find that the previous functional regions of the cerebellum can mainly divide into the “motor” cerebellum (comprising lobules V, VI, VIIb, and VIII) and the “cognitive” cerebellum (comprising Crus I and II).^122^ However, according to the existing research, the “motor” cerebellum and the “cognitive” cerebellum are not as distinct as previously mentioned but rather as a state of mutual integration (Figure 1). This may be due to the complex FCs between the internal structures of the cerebellum. This further provides an anatomic basis for the cerebellum to play an important involvement in nonmotor functions. Therefore, further exploration of pathological and biochemical changes in the AD cerebellum may provide a basis for a better understanding of the contribution of the cerebellum in the clinical manifestations of AD.

**TABLE 1:** Overlapping clinical manifestations of Alzheimer's disease (AD), vascular dementia (VaD), Lewy body dementia (DLB), frontotemporal dementia (FTD), Parkinson's disease (PD), amyotrophic lateral sclerosis (ALS), Huntington's disease (HD), and spinocerebellar ataxias (SCA) in association with cerebellar dysfunctions

Symptoms	AD	VaD	DLB	FTD	PD	ALS	HD	SCA	Cerebellar dysfunction
Cognitive impairments	●	●	●	●	⦿	⦿	⦿	⦿	⦿
Memory impairment	●	●	●	●	⦿	⦿	⦿	⊙	⦿
Executive dysfunction	●	●	●	●	●	⦿	⦿	⊙	⦿
Visual‐spatial disorder	●	⦿	⊙	⊙	⊙	○	⊙	○	⊙
Changes in personality, behavior, or demeanor	●	⊙	○	●	⦿	⦿	⊙	○	⦿
Language disorder	●	●	●	●	●	⦿	⊙	●	⦿
Sleep disorder	●	●	●	●	●	⦿	⊙	⦿	⦿
Mood disorder	●	●	●	●	●	⊙	⦿	⦿	⦿
Autonomic dysfunction	●	⦿	●	●	●	⊙	⊙	⦿	⦿
Sensory impairments	●	⦿	⦿	●	●	⦿	⦿	⊙	⦿
Olfactory dysfunction	⦿	⊙	⦿	⦿	⦿	⊙	⊙	⊙	⦿
Hearing impairment	⦿	⊙	⊙	⦿	⦿	⊙	⊙	⊙	⦿
Visual impairment	⦿	⦿	⊙	○	⦿	⊙	⊙	⊙	⦿
Frailty	⊙	⦿	⊙	○	●	○	○	○	⊙
Pain	⊙	⦿	⊙	⦿	●	●	●	⊙	⊙
Motor dysfunctions	⦿	●	●	●	●	●	●	●	●
Dyskinesia	⦿	⊙	⊙	⊙	●	⊙	●	⦿	●
Tremor	⦿	⊙	⦿	●	●	⦿	⦿	⦿	●
Dystonia	⊙	⦿	⊙	⊙	●	⊙	⦿	●	●
Voluntary movement disorder	⊙	●	○	○	⊙	●	⦿	○	⦿
Involuntary movement disorder	⦿	⊙	●	⊙	●	○	●	●	●

○No evidence ⊙Weak evidence ⦿Moderate evidence ●Strong evidence.

Abbreviations: AD, Alzheimer's disease; ALS, amyotrophic lateral sclerosis; DLB, Lewy body dementia; FTD, frontotemporal dementia; HD, Huntington's disease; PD, Parkinson's disease; SCA, spinocerebellar ataxias; VaD, vascular dementia.

### 2.2 The cerebellum may participate in the pathological and biochemical changes of AD

**FIGURE 2:**
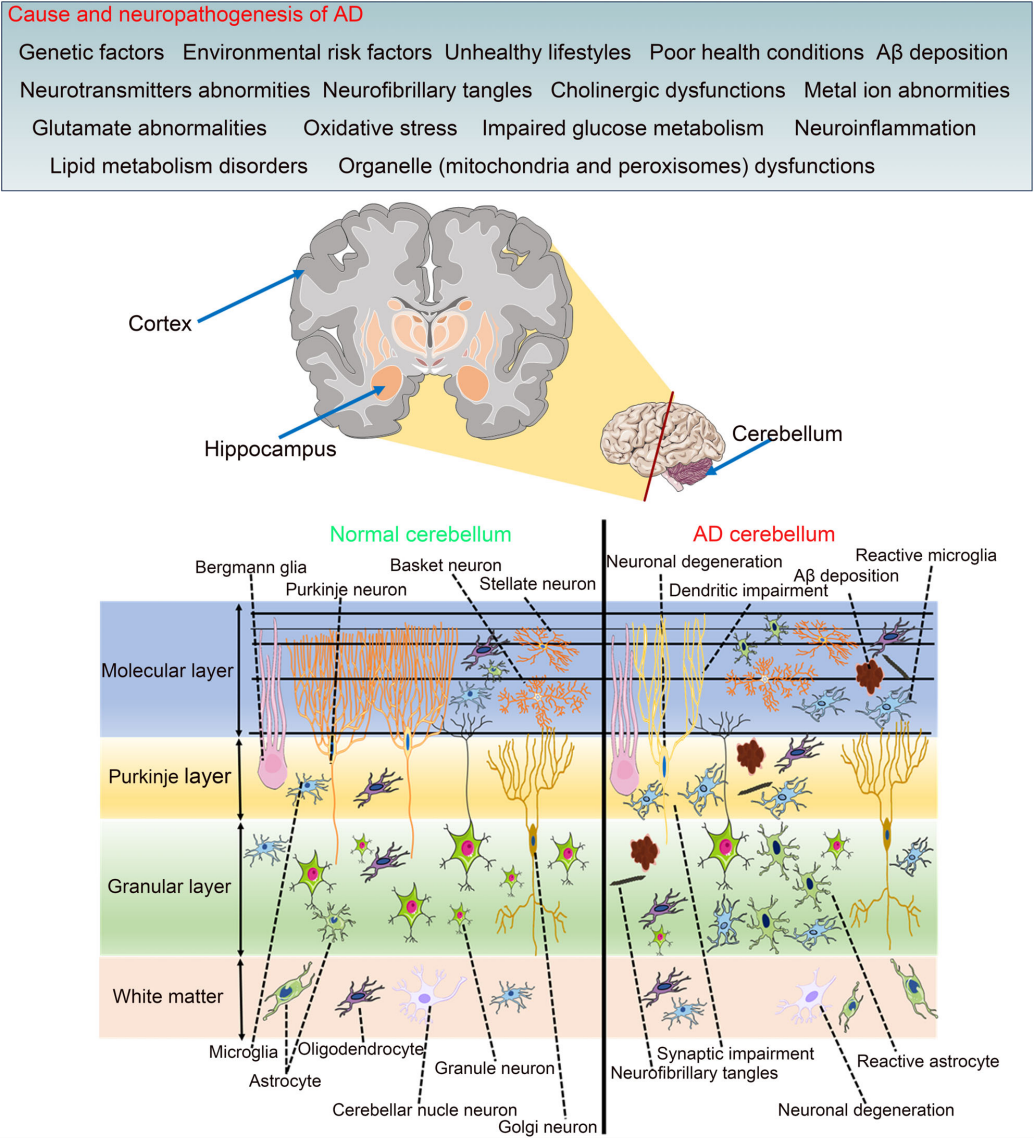
Cause and neuropathogenesis of cortex/hippocampus and pathological changes of cerebellum in AD. Pathogenesis of cortex/hippocampus includes genetic factors, environmental risk factors, unhealthy lifestyles, poor health conditions, Aβ deposition, neurotransmitter abnormities, neurofibrillary tangles, cholinergic dysfunctions, metal ion abnormities, glutamate abnormalities, oxidative stress, impaired glucose metabolism, neuroinflammation, lipid metabolism disorders, and organelle (mitochondria and peroxisomes) dysfunctions. Schematic graph showing cerebellar cytoarchitecture. The cerebellar cortex is composed of three layers, from the outside to the inside is divided into the molecular layer, Purkinje layer, and granular layer. The cerebellar cortex comprises five distinct types of neurons: stellate neurons and basket neurons in the molecular layer, Purkinje neurons in the Purkinje layer, and granule neurons and Golgi neurons in the granular layer. The cerebellar white matter comprises cerebellar nuclei neurons. There are three types of glial cells in the cerebellum: astrocytes in the molecular layer, granular layer and white matter, microglia and oligodendrocytes in all layers and white matter. Bergmann glia are specialized astrocytes in the cerebellar cortex, their cell bodies are located in the Purkinje layer, and the dendrites and fibers pass upward through the molecular layer. The cerebellum of AD also showed pathological changes, including the appearance of Aβ deposition and neurofibrillary tangles, a decrease in the number of neurons, the abnormality of the structure and function of synapses and dendrites, and abnormal neuroinflammatory. AD, Alzheimer's disease; Aβ, amyloid‐β.

The core pathogenesis of AD involves amyloid‐β (Aβ) deposition, hyperphosphorylated tau (p‐Tau), neuroinflammation, oxidative stress,^123^, ^124^ and others (Figure 2). Recent research highlights the cerebellum's involvement in the disease's pathogenesis.

Pathological hallmarks of AD encompass Aβ deposition, p‐Tau, neuronal damage, and inflammatory responses.^125^ Historically, research focus has been on the cortex and hippocampus, with the cerebellum considered exempt from these changes.^17^ However, recent findings reveal that the cerebellum in AD exhibits comparable pathologies to the cortex and hippocampus, such as Aβ deposition,^126^, ^127^, ^128^, ^129^ p‐Tau,^130^ neuron loss,^131^, ^132^, ^133^ synaptic and dendritic abnormalities,^134^, ^135^, ^136^ and augmented neuroinflammation (Figure 2).^137^, ^138^ Alongside these pathologies, biochemical alterations with oxidative stress,^139^, ^140^ metabolic changes, and neurotransmitter abnormalities are evident in the cerebellum of both AD patients and animal models.^141^, ^142^, ^143^, ^144^

#### 2.2.1 Aβ deposition and p‐Tau in the cerebellum of AD

Aβ deposition and p‐Tau are central molecules in the pathogenesis of AD.^145^ However, research predominantly focuses on Aβ plaques and neurofibrillary tangles in the cortex and hippocampus, with conflicting reports on their presence in the cerebellum.^111^, ^126^, ^128^

In early neuropathological investigations, Aβ plaques and neurofibrillary changes are typically undetected in the cerebellum of AD patients. One study has reported a limited number of diffuse plaques in the cerebellar cortex, significantly fewer than those found in the prefrontal and parietal cortices.^146^ Nevertheless, more recent studies have shown that Aβ is indeed present in the cerebellum in AD cases,^128^, ^147^, ^148^, ^149^ albeit at a lower proportion than in the medial temporal cortex.^126^ Notably, all previous studies are notably after clinical onset; then, before clinical onset, it is not known whether there is also Aβ plaque deposition in the cerebellum. A study has reported cerebellar Aβ plaque deposition in cognitively unimpaired carriers starting about a decade before the clinical onset of autosomal dominant AD.^150^ Furthermore, p‐Tau has been detected in the cerebellum of AD patients.^130^ One study has identified p‐Tau deposition in the cerebellum of patients with PS1‐E280A patients.^151^ Another study has reported p‐Tau accumulation in the cerebellum of AD patients; however, it is worth noticing that the level of p‐Tau does not increase as the disease progresses.^148^

To delve deeper into the distribution and progression of Aβ plaques in the cerebellum of AD, various animal model studies have been conducted. However, the results are significantly different. For instance, in APP/PS1 mice, Aβ40/Aβ42 also is detected at the age of 2 and 3 months by enzyme‐linked immunosorbent assay in the cerebellum.^8^, ^152^, ^153^ Cerebellar Aβ plaques become detectable at 6 months of age and gradually increase as the mice age in APP/PS1 mice.^129^, ^154^ Nonetheless, other literature has suggested that cerebellar Aβ plaques may not be detectable until after the age of 8 months in APP/PS1 mice.^8^, ^153^, ^155^, ^156^, ^157^, ^158^ Additionally, in 9‐month‐old 5xFAD mice, the cerebellum has exhibited low levels of Aβ plaques.^159^ On the other hand, the severity of Aβ plaque deposition in the cerebellum is influenced by the mouse gender, with females displaying higher occurrence rates compared with males.^154^ Aβ plaques are mainly localized in the Purkinje cell (PC) layer,^160^ molecular layers, and granule cell layer of the cerebellum.^158^

#### 2.2.2 Neuronal degeneration in the cerebellum of AD

Numerous neurons undergo degeneration and cell death in AD.^161^ This raises the question of whether neuronal degeneration also occurs in the cerebellum of AD patients. Although some studies have reported no significant difference in the density of PCs or granule cells between AD and normal control brains,^131^ others have shown significant damage to PCs and granule cells in the cerebellar cortex in AD. For instance, Fukutani et al.^162^ found a significantly decreased density of PCs in both familial and sporadic AD cases. Additionally, several studies have reported a substantial reduction in the number of PCs in the cerebellar vermis and the superior surface of the cerebellar hemispheres in AD patients.^134^, ^146^ Wegiel et al.^163^ also reported a loss of PCs and granule cells in AD. Besides, the morphology of the Golgi apparatus of the cerebellar PCs is abnormal in AD patients.^164^

Significant neuronal loss is also found in the cerebellum of animal models of AD.^132^, ^133^ In APP/PS1 mice, a strong trend toward loss of PCs is found at the age of 5 months.^129^ Compared with wild‐type (WT) mice, APP/PS1 mice have 18 and 25% lower linear cell densities of PCs at 12 and 18/20 months of age, respectively.^158^ In APP transgenic mice, the number of PCs at 10 months of age is about 60% of that in WT mice and decreases progressively with age.^132^ Notably, in 3xTg‐AD mice, neurons in the deep cerebellar nucleus appear vulnerable even in the early stage of AD.^133^

Further, in the cerebellum of AD animal models, not only the number of neurons is reduced, but also the morphological structure of neurons is altered. In APP/PS1 mice, PCs exhibit various pathological changes, such as cytoplasmic atrophy, nuclear darkening, and axon enlargement.^158^

#### 2.2.3 Synapse and dendrite impairments in the cerebellum of AD

Synaptic loss is a primary pathophysiological marker in AD.^165^ Researchers have actively investigated whether synaptic abnormalities occur in the cerebellum of AD patients. Studies using TgCRND8 mice have demonstrated early‐stage synaptic plasticity defects in the cerebellar brain.^166^ Similarly, synaptic impairments have been reported in the cerebellum of 7–8‐month‐old APP/PS1 mice.^152^

Dendritic damage and diminished spine density are profound features in the early stages of AD.^167^, ^168^ AD is characterized by the loss of distal dendritic segments and altered spine morphology, specifically in the PCs of the cerebellar hemisphere's inferior surface.^134^ Mavroudis et al.^134^, ^146^ have observed a 50% decrease in dendritic spine density of the cerebellar vermis's PCs and a 43% reduction in the anterior lobe of AD brains. Additionally, they have noted a potential reconstruction of dendritic spines, with most remaining spines being short and stubby, contrasting the higher proportion of long‐necked spines in normal controls. Notably, AD brains exhibit numerous filopodia and dystrophic dendritic spines in PCs.^134^, ^146^

#### 2.2.4 Neuroinflammation in the cerebellum of AD

Growing evidence implicates glial‐cell‐mediated neuroinflammatory processes in the progression of AD, with microglia and astrocytes playing crucial roles in innate immune responses to disease stressors.^169^ One study has revealed a 91% increase in microglia numbers within the cerebellum of AD patients.^170^ It has been reported that the cerebellar astrocyte count in AD patients is 11 times higher than in healthy controls.^138^ Further studies have exposed significant astrocyte morphological and immunoreactive subtype alterations in the aged and AD cerebellum.^171^ Moreover, rapidly progressive AD cases have exhibited a distinct microglial phenotype in the cerebellum.^172^

The upregulation of microglia and astrocyte markers in the cerebellum of AD model animals has been well documented. In APP/PS1 mice, astrocyte aggregates and microglial responses around Aβ plaques have been observed in the granular layer, with microglial responses surrounding Aβ plaques in the molecular layer. Quantitative analysis has indicated fewer activated microglia around Aβ plaques in the cerebellum of APP/PS1 mice.^153^ Additionally, the molecular layer of APP/PS1 mice has exhibited an augmented number of astrocytes compared with age‐matched controls.^158^ Another study has reported a glial reaction confined to the granular layer of 15‐month‐old APP/PS1 mice.^154^

#### 2.2.5 Oxidative stress and mitochondrial dysfunctions in the cerebellum of AD

Oxidative stress is a well‐known risk factor for AD.^123^, ^173^ Prior research has revealed the elevated lipid peroxidation levels and the presence of neurotoxic lipid peroxidation byproducts, such as 4‐hydroxynonenal (HNE) and acrolein, in the AD brain.^173^ This study further has shown a significantly increased level of HNE in the cerebellum of MCI and elevated levels of acrolein in the early AD.^173^ Moreover, oxidative stress abnormalities have been observed in the cerebellum of AD animal models.^139^

Meanwhile, mitochondrial dysfunction is a characteristic of AD.^174^, ^175^ Research has shown that this dysfunction is also present in the cerebellum of AD patients, including the altered expression of genes associated with mitochondrial biogenesis.^176^ Notably, mitochondrial abnormalities have been reported in the cerebellum of PS1‐E280A patients^128^ and 5xFAD transgenic mice at 7 weeks of age, with downregulated genes related to mitochondrial dysfunction in the cerebellum.^144^ Another study also found abnormal mitochondria in APP/PS1 mice aged 18/20 months.^158^

#### 2.2.6 Metabolic changes in the cerebellum of AD

Cholesterol metabolic disorders are strongly linked to AD progression. In 9‐month‐old APP/PS1 mice, as AD‐like pathology started to develop, there was an elevation of cholesterol precursors desmosterol and 27‐hydroxy(OH)cholesterol in the cerebellum.^141^ At the age of 21 months, 24(S)‐OH‐cholesterol level also is increased in the cerebellum of the APP/PS1 mice.^141^

Additionally, other metabolic disturbances have been identified in the AD cerebellum. Metabolomic analysis of 5‐month‐old APP/PS1 mice has revealed disturbed energy metabolism and amino acid metabolism in the cerebellum and cortex.^142^ Nuclear magnetic resonance spectral analysis has highlighted metabolic disruptions in the cerebellum of TgCRND8 mice.^143^ Furthermore, proteomics studies have identified 34 proteins with differential expression in the AD cerebellum compared with controls.^177^

#### 2.2.7 Neurotransmitters and receptors abnormalities in the cerebellum of AD

Dysregulation of cholinergic and adrenergic systems is associated with AD.^178^ Postmortem analyses of AD brains have demonstrated a loss of cholinergic function and markers in the cortex.^179^ Further, a study also has suggested that the cerebellum may have a direct influence on the cholinergic dysfunction in AD.^180^ Choline acetyltransferase activity is significantly decreased in the cerebellum of early‐onset AD patients compared with controls,^181^ and high acetylation level is observed in the AD cerebellum.^182^ Regarding the noradrenaline system, the cerebellum of AD patients has exhibited abnormalities, including reduced noradtenaline levels in TgCRND8 mice.^183^

#### 2.2.8 Genes and ribosomal RNA changes in the cerebellum of AD

During AD onset, abnormal gene and ribosomal RNA (rRNA) expression have been implicated in the pathogenesis.^184^ There are significant differences in the expression of key genes in the cerebellum between AD and controls.^185^ A study has found differential hypermethylation upstream of the ribosomal RNA human genes (rDNA) promoter in the cerebellum compared with the auditory cortex, suggesting a regulatory region for rDNA expression. Furthermore, comparing AD and control cerebellum samples, researchers have detected hypermethylation of the rDNA promoter and increased rDNA content.^184^ Additionally, rRNA levels have been augmented in the AD cerebellum compared with control samples.^184^

Both AD patients and animal models exhibit similar neuropathological changes in the cerebellum, comparable to the cortex and hippocampus (Tables 2 and 3), indicating its involvement in AD progression.

**TABLE 2:** Comparison of pathological and biochemical alterations of cerebellum, cortex, and/or hippocampus in Alzheimer's disease/mild cognitive impairment patients.

Disease	Pathology and biochemistry	Cerebellum	Cortex or/and hippocampus	References
AD	Aβ	Aβ was observed.	Aβ of the same cases in far bigger proportion than cerebellum.	^126^, ^146^, ^149^
	P‐Tau	P‐Tau was observed.	High p‐Tau was found.	^148^, ^151^
	Neuron damage	A loss of PCs and a marked decrease in the density of dendritic arborization were observed.	A reduced density of Pyramidal cells on the frontal cortex was found.	^146^
	Neuroinflammatory damage	Microglial proteins Iba1 was increased.	Microglial proteins Iba1 was not increased but IL‐15 was increased in the temporal cortex.	^148^
		Microglia and astrocytes were observed.	An elevated number of microglia and astrocytes were observed.	^172^
	Mitochondrial abnormalities	Dysregulated genes involved in mitochondrial cellular biosynthesis were found.	In the frontal cortex, genes involved in mitochondrial energy, ATP, and oxidative phosphorylation, were the most significant dysregulated genes.	^176^
	H3K9 acetylation changes	A hyperacetylation was found.	A slight hypoacetylation of the hippocampus was found.	^182^
	Gene changes	The differences in the expressions of FCGRT, SLC1A3, PTN, PTPRZ1, and PON2s among the AD and control groups were significant.	The differences in the expressions of FCGRT, SLC1A3, PTN, PTPRZ1, and PON2 in the frontal cortex tissues among the AD and control groups were significant.	^185^
	Lipid metabolism abnormality	An increase in acrolein was found.	An increase in HNE and acrolein in HPG and SMTG was found.	^173^
MCI	Lipid metabolism abnormality	An increase in HNE was found.	An increase in HNE in HPG and SMTG was found.	^173^

Abbreviations: Aβ, amyloid‐β; AD, Alzheimer's disease; ATP, adenosine triphosphate; FCGRT, Fc gamma receptor and transporter; H3K9, histone 3 lysine 9; HNE, hydroxynonenal; HPG, hippocampus/parahippocampal gyrus; Iba1, ionized calcium‐binding adapter molecule 1; IL‐15, interleukin‐15; MCI, mild cognitive impairment; PC, Purkinje cell; P‐Tau, hyperphosphorylated tau; PON2, paraoxonase 2; PTN, pleiotrophin; PTPRZ1, protein tyrosine phosphatase receptor type Z1; SLC1A3, solute carrier family 1 member 3; SMTG, superior and middle temporal gyrus.

**TABLE 3:** Comparison of pathological and biochemical changes of cerebellum, cortex and/or hippocampus in animal models of Alzheimer's disease.

Animal models of AD	Pathology and biochemistry	Cerebellum	Cortex or/and hippocampus	References
APP/PS1 mice	Aβ	Aβ was observed.	Aβ appears earlier than the cerebellum.	^8^
		Aβ was observed.	A higher Aβ load was detected.	^129^, ^153^
		Soluble Aβ42 was observed.	At 2 months of age, the level of soluble Aβ42 in the forebrain was higher than that in the cerebellum; at 8 months of age, the level of soluble Aβ42 in the forebrain was lower than that in the cerebellum.	^152^
	Neuroinflammatory damage	Iba1 was observed.	The level of iba1 expression in the cerebral cortex and hippocampus was higher than that in the cerebellum.	^153^
	Lipid metabolism abnormality	24(S)‐OHcholesterol and 27‐OHcholesterol levels were increased.	24(S)‐OHcholesterol levels were increased.	^141^
	Metabolism abnormality	The observation of extensive metabolic alterations was observed.	The observation of extensive metabolic alterations was observed in the cortex, but not in the hippocampus.	^142^
5xFAD mice	Aβ	Aβ was observed.	A higher Aβ load was detected.	^159^
	Gene changes	Mitochondrial dysfunction‐related genes were evident in downregulated DEGs.	Cardiovascular disease‐related genes were found in downregulated DEGs of the frontal cortex.	^144^
TgCRND8 mice	Metabolism abnormality	The perturbations in metabolism were more widespread.	The perturbations in metabolism have been most affected in the cortex and hippocampus.	^143^
	Noradrenaline change	Reductions in noradrenaline were observed.	Reductions in noradrenaline were observed.	^183^

Abbreviations: Aβ, amyloid‐β; AD, Alzheimer's disease; DEGs, differentially expressed genes; Iba1, ionized calcium‐binding adapter molecule 1.

Several studies have shown that lesions in the cerebellum appear later or to a lesser extent than in the cortex and hippocampus (Tables 2 and 3). This may be because the cerebellum may be resistant to certain neurodegenerative mechanisms. Compared with other parts of the brain, cerebellar accumulation of mitochondrial DNA deletions, oxidative damage to mitochondrial and nuclear DNA, changes in gene expression, metabolic and oxidative stress markers, silencing message regulator 1 and superoxide dismutase 1, and age‐related DNA methylation levels are all low.^186^

Besides, one study has shown that in the early stages of the disease, damage to the cerebellum is less severe than that of the cortex and hippocampus. However, in the advanced stage of the disease, the cerebellum is more severely damaged than the cortex and hippocampus (Tables 2 and 3). Therefore, the cerebellum may be an early warning sign of disease progression in AD.

The exploration of whether there's a temporal and/or spatial correlation between cerebellar lesions and abnormalities in the cortex and hippocampus warrants further investigation because the bulk of existing research is skewed towards the cortex and hippocampus, relegating cerebellar studies in AD to isolated observations. There's a notable scarcity of studies examining the cortex and hippocampus alongside the cerebellum across identical disease stages. Moreover, current investigations into the cerebellum are predominantly confined to phenomenological observations, leaving the deeper molecular mechanisms largely unexplored. For instance, while Aβ plaques are identified in the AD cerebellum, reports on the associated metabolic enzymes are absent. Similarly, mitochondrial dysfunction is recognized within the AD cerebellum without detailed insights into its underlying mechanisms.

A more meticulous analysis of the structural and functional changes within the AD cerebellum could illuminate the damage of brain tissue prompted by pathological and biochemical shifts in the cerebellum. Such insights would significantly enhance our understanding of the cerebellum's contribution to the clinical manifestations of AD.

### 2.3 Imaging changes in the cerebellum of AD

As imaging continues to evolve, imaging can provide information not only on structural changes in tissues but also on tissue function for research. Existing studies have also shown that in addition to the above pathophysiological changes, imaging studies including magnetic resonance imaging (MRI) and functional MRI provide further evidence supporting the involvement of the cerebellum in AD.

#### 2.3.1 Structure imaging changes in the cerebellum of AD

Cerebellar atrophy is a common feature in AD patients, with the degree and location of atrophy varying among individuals.^187^ In some AD patients, the right cerebellum experiences significant atrophy,^188^ whereas in others, the volume of the cerebellum hemisphere, posterior cerebellum, and vermis is reduced.^189^ The timing of cerebellar atrophy occurrence in AD patients also varies across studies. Some studies have indicated that cerebellar atrophy primarily manifests in the late stage of AD,^190^ whereas others have suggested that changes in the cerebellar cortex volume persist throughout the early to advanced clinical stages of AD. Notably, the vermis and para‐vermian areas of the anterior (I–V) and posterior (VI) lobes are affected as early as the amnestic MCI (aMCI) stage, with later involvement of the hemispheric part of the posterior lobes (VI lobule) and Crus I specifically in AD patients.^187^

#### 2.3.2 Functional imaging changes in the cerebellum of AD

Previous studies have consistently reported disrupted FC in various brain networks of patients with aMCI and AD.^191^, ^192^, ^193^ Specifically, a study has demonstrated distinct patterns of FC between cerebellar sub‐regions and diverse functional networks, which are differentially impaired in AD patients.^191^ Additionally, other studies have revealed alterations in cerebellar FC with cerebral cortical regions, as well as the correlation between cerebellar FC and cognitive impairment in AD and aMCI.^192^, ^194^ Compared with healthy controls, aMCI patients have exhibited significantly lower hippocampal FC within a network involving the cerebellum.^195^ These findings have suggested that abnormalities in cerebellar FC may serve as a sensitive and preferable indicator of dysfunction than local activity measures in aMCI patients.^196^ In summary, there is compelling evidence of aberrant FC in the cerebellum of AD patients. Furthermore, the specific location of these abnormal FC patterns has been linked to different clinical manifestations.^197^ For instance, disrupted FC between the left Crus II and the right thalamus, as well as between the left lobule IX and the right parietal lobe, is both associated with cognitive decline in AD. Similarly, disrupted FC between the left Crus II and the right thalamus, as well as between the left lobule IX and the right parietal lobe, has been associated with attention deficits among subjects with MCI.^197^

### 2.4 Electrophysiological changes in the cerebellum of AD

Abnormal electroencephalography (EEG) markers are associated with cognitive dysfunction in AD patients.^198^ However, at present, the electrophysiological changes have primarily focused on the cerebral cortex,^199^ while the electrophysiological alterations in the cerebellum of AD patients remain in the preliminary exploration stage. The apolipoprotein‐E (APOE) ε4 allele is the greatest genetic risk factor for late‐onset AD, and abnormalities in the cerebellar EEG are correlated with the cognitive impact of ε4 carriers (ε4+).^200^ This suggests that there may be abnormal cerebellar function even in the early clinical stage of preclinical AD. Previous studies conducted by our research team have also shown significant cerebellar EEG changes in APP/PS1 mouse models.^8^ Furthermore, these alterations in the cerebellar EEG and sleep–wake cycles have been observed before the onset of cognitive impairment and the development of new pathological hallmarks of AD.^8^ Based on these findings, we hypothesize that the electrophysiological features of the cerebellum during the sleep–wake cycle could serve as potential markers for the prepathologic detection of AD.^8^ Interestingly, other research teams have also reported widespread signs of electrophysiological changes in PCs and cerebellar deep nucleus neurons in APP/PS1 mice.^201^ Specifically, altered neuronal firing in the PC layer of APP/PS1 mice is accompanied by dual local field potential oscillations.^201^

Neuroimaging is a noninvasive and repeatable method that can provide comprehensive three‐dimensional structural and functional information about the brain. In contrast, electrophysiology measures real‐time changes in neural activity and can offer functional insights. Combining these complementary approaches could help researchers a more comprehensive understanding of neural processes. Studies utilizing both neuroimaging and electrophysiology have demonstrated that there are changes in the cerebellum of individuals with AD, indicating that the cerebellum should not be overlooked in AD research. However, the specific pattern of cerebellar changes observed in AD can vary across different experiments, possibly due to the heterogeneity of subject populations. This suggests the need for larger sample sizes and more detailed sample classifications, such as stratification based on disease progression or clinical manifestations, to further investigate cerebellar alterations in AD. Overall, identifying specific patterns of cerebellar changes using both neuroimaging and electrophysiological techniques in different subtypes of AD can help guide clinical diagnosis and inform the development of targeted treatment strategies.

### 2.5 The cerebellum may be a therapeutic target for AD

Although no studies have yet elucidated the specific role of the cerebellum in the pathogenesis and progression of AD, several important questions remain unanswered. For instance, it is unclear whether the pathological, neuroimaging, and electrophysiological changes observed in the cerebellum are temporally and spatially related to the changes occurring in the cerebral cortex and hippocampus. Additionally, it is unknown whether the alterations in the cerebellum directly map onto the clinical signs and symptoms of AD. However, the existing body of evidence has consistently demonstrated that the cerebellum is indeed involved in the disease process of AD. Interestingly, the cerebellum has emerged as a promising target for neurostimulation therapies in various neurological disorders. Current neuromodulation approaches being investigated for the treatment of AD include transcranial magnetic stimulation (TMS), theta burst stimulation (TBS), and deep brain stimulation (DBS).^202^ Whether cerebellar TMS, TBS, and DBS can improve cognitive function is also under investigation. Studies have demonstrated that the cerebellar rTMS treatments can result in significant improvements in multidomain cognitive function, along with enhancing brain connectivity in AD patients.^20^ Besides, studies have shown that short‐latency afferent inhibition (SLAI) is reduced in AD patients compared with controls, while cerebellar continuous TBS partially restore SLAI in AD patients.^180^ Finally, DBS has been used in PD and other motor disorders, but cerebellar DBS still needs to be evaluated in AD. Besides, a study shows that exercise training can improve PC survival.^203^

In addition to electrical stimulation, pharmacological interventions targeting the cerebellum have also shown promise in AD. For example, the natural compounds naringin and hesperidin have been found to ameliorate oxidative stress in the cerebellum of AD models.^139^, ^140^ Similarly, prolactin‐releasing peptides can reduce Aβ plaques and microgliosis in the cerebellum. Furthermore, the palmatine has demonstrated the potential to improve cognitive function in a transgenic mouse model of AD, with the most prominent effects observed in the cerebellum, followed by the hippocampus, but not the cerebral cortex.^204^ These findings collectively suggest that the cerebellum may be a valuable therapeutic target for the development of novel pharmacological strategies in AD.

Cerebellar neuroscience has experienced a paradigm shift in recent years. Advances in understanding the brain‐cerebellar interplay, organizational principles of cerebral cortical connections, neuroanatomy, clinical observations, and neuroimaging have collectively positioned the cerebellum as a critical component within the distributed neural circuits displaying nonmotor function. The proper functioning of the brain‐cerebellar circuit is essential for both cognitive and noncognitive functions. Some damage may interfere with the normal function of this circuit, leading to the appearance of cognitive and noncognitive dysfunction. AD cerebellum has the same AD‐like pathological changes as the hippocampus and cortex, including the appearance of Aβ plaques, the loss of neurons, the abnormality of the structure and function of synapses and dendrites, and the increased neuroinflammatory response. In addition, there are abnormal biochemical changes in the AD cerebellum. Neuroimaging and electrophysiology have also further confirmed the abnormality of cerebellar structure and function in AD. These neuropathologies may result in structural and functional abnormalities of the brain‐cerebellar circuit, further leading to cognitive impairment, sleep disturbances, mood disorders, autonomic dysfunction, and specific sensory disorders (olfaction, hearing, vision) and others.

Therefore, we believe that the cerebellum is more than a mere passive observer in the pathophysiology and clinical manifestation of AD. Certainly, further research is required to elucidate the temporal and spatial stages of AD pathology in the cerebellum, how cerebellar interactions affect brain connectivity, and how their changes impact cognitive performance and noncognitive function in AD. This exploration may pave the way for new therapeutic opportunities for AD.

## 3 CEREBELLUM AND OTHER DEMENTIAS

AD is the most common cause of dementia, followed by dementia with VaD, DLB, and FTD.^205^ The cerebellum appears to play an important role in AD, but the role of the cerebellum in other types of dementia remains unclear.

### 3.1 Cerebellum and VaD

VaD is the second most common form of dementia and is caused by vascular pathologies.^206^ Cerebellar stroke can cause cognitive impairment.^19^ Notably, the cerebellum shares extensive and bidirectional connections with a large portion of the area in the brain. That is, stroke in any part of the brain could influence the cerebellum. Vascular pathologies in the cerebellum not only cause neuronal and synaptic damage,^207^ neurotransmitter and receptor abnormalities,^208^ but also mitochondrial and oxidative stress abnormalities,^209^ as well as metabolic and gene changes.^210^, ^211^

A study has noted abnormal nuclei shapes in PCs in the vermis region, with a statistically significant reduction in the mean total number of PCs in a mouse model of VaD compared with control animals.^212^ Moreover, another study has identified apoptosis and pyroptosis in the cerebellum in a mouse model of VaD.^206^ Imaging studies have suggested that white matter changes in the cerebellum may be linked to executive function and memory performance in patients with subcortical vascular MCI.^213^ Furthermore, therapeutic stimulation of the cerebellar fastigial nucleus has shown beneficial effects in VaD treatment.^214^ Hence, the cerebellum may play a role in the development of VaD.

### 3.2 Cerebellum and DLB

DLB is the second most common cause of neurodegenerative dementia after AD.^215^ The clinical manifestations of DLB revolve around visuospatial, executive, and attention deficit recognition. These cognitive symptoms, along with Parkinson's syndrome, cognitive fluctuations, hallucinations, and REM sleep behavior disorder, are central features of DLB.^215^

The cerebellum does not appear to be the focus of DLB in conventional cognition, but it has also been shown that there is pathological damage to the cerebellum in DLB. Notably, a study has identified numerous alpha‐synuclein‐positive, round inclusions within the cerebellar white matter of DLB patients, predominantly situated in PC axons.^216^ Additionally, widespread epigenetic dysregulation in genes associated with neuron‐to‐neuron synapse, postsynaptic specialization, and postsynaptic density in the cerebellum of DLB.^217^ Metabolic dysregulation,^218^ decreased APOE DNA methylation,^219^ and abnormal cerebellar noradrenergic system functioning further distinguish DLB cerebellar pathology from controls.^220^ Structural alterations, such as cerebellar gray matter loss in the left Crus I and right Crus II regions, have been documented in DLB cases,^221^ with associated impairments in writing function linked to reduced gray matter in the right cerebellum among early‐stage DLB patients.^222^ Moreover, DLB has exhibited disrupted connectivity within cerebellar networks compared with controls,^223^, ^224^, ^225^ with hypermetabolism predominantly localized in the cerebellum.^226^, ^227^ In conclusion, a comprehensive investigation of the cerebellum is warranted in DLB research, as further exploration may unveil novel avenues for both prevention and treatment strategies.

### 3.3 Cerebellum and FTD

FTD stands as a prevalent form of young‐onset dementia, constituting a neurodegenerative condition primarily impacting the frontal and/or temporal lobes.^228^

FTD categorization includes two main types: sporadic and genetic FTD, delineated by etiology, and further subdivided into behavioral variant FTD (bvFTD), semantic dementia (SD), and progressive nonfluent aphasia (PNFA) based on clinical presentation.^229^ Approximately one‐third of FTD patients carry an autosomal dominant mutation in one of three genes: microtubule‐associated protein tau (MAPT), progranulin, and chromosome 9 open reading frame 72 (C9orf72).^230^

Although historically the cerebellum has been considered unrelated to FTD pathology, emerging research indicates its significant involvement in the neurodegenerative process.^231^ A study has noted downregulation of neuronal markers in the cerebellum alongside upregulation of microglia, astrocytes, oligodendrocytes, endothelial cells, and pericytes in FTLD‐TDP patients.^232^ Additionally, dendritic arborization defects, dysregulated synaptic genes, and altered neurotransmission contribute to cerebellar synaptic dysfunction in C9orf72‐associated ALS (C9‐ALS)/FTD.^233^ Moreover, the unfolded protein response and casein kinase 1 delta involvement have been observed in C9‐FTD, with increased presence noted in granule cells within the cerebellum.^234^ Furthermore, toxicity from dipeptide repeat proteins (DPR) underscores one of the proposed pathogenesis of FTD, with the cerebellum exhibiting both the highest DPR load and relative DPR solubility.^235^ Last, cerebellar differentially expressed genes have been identified in FTD.^236^ Different FTD subtypes and AD share similar metabolic phenotypes in the cerebellum.^237^

Imaging studies have also revealed cerebellar structural and functional abnormalities in FTD, with subtype‐specific patterns evident.^230^, ^238^ Notably, C9ORF72 mutation carriers have exhibited greater atrophy rates in the left cerebellum and right occipital lobe compared with MAPT carriers, whereas sporadic FTD patients have demonstrated greater anterior cingulate atrophy compared with C9ORF72 and MAPT carriers.^239^ Moreover, cerebellar atrophy correlates solely with anxiety in C9orf72 carriers.^240^ Distinct pathological patterns in the cerebellum have been observed among bvFTD, SD, and PNFA,^241^, ^242^, ^243^ with bvFTD exhibiting greater cerebellar white matter changes compared with SD and PNFA.^229^

Taken together, these findings suggest a nuanced role for the cerebellum in FTD, potentially serving as a marker for subtype differentiation. Further exploration of the cerebellum‐FTD relationship promises a deeper understanding of FTD's pathological mechanisms.

## 4 CEREBELLUM AND OTHER NDs

The cerebellum has emerged as a new frontier for research in AD and other dementias. Cerebellar dysfunction is also becoming a prominent theme in other NDs, including PD,^122^ ALS, HD, and SCA. In this review, we explore the role of the cerebellum in the pathogenesis of various NDs and highlight the cerebellar vulnerabilities that may contribute to the development of these disorders.

### 4.1 Cerebellum and PD

PD is one of the most common NDs.^244^ Until recently, the role of the cerebellum in PD has received relatively little attention. There is growing recognition that cerebellar involvement may explain some of the motor and nonmotor symptoms associated with PD (Table 1).^122^ Furthermore, studies have demonstrated pathophysiological and imaging changes in the cerebellum of PD patients.^122^

**FIGURE 3:**
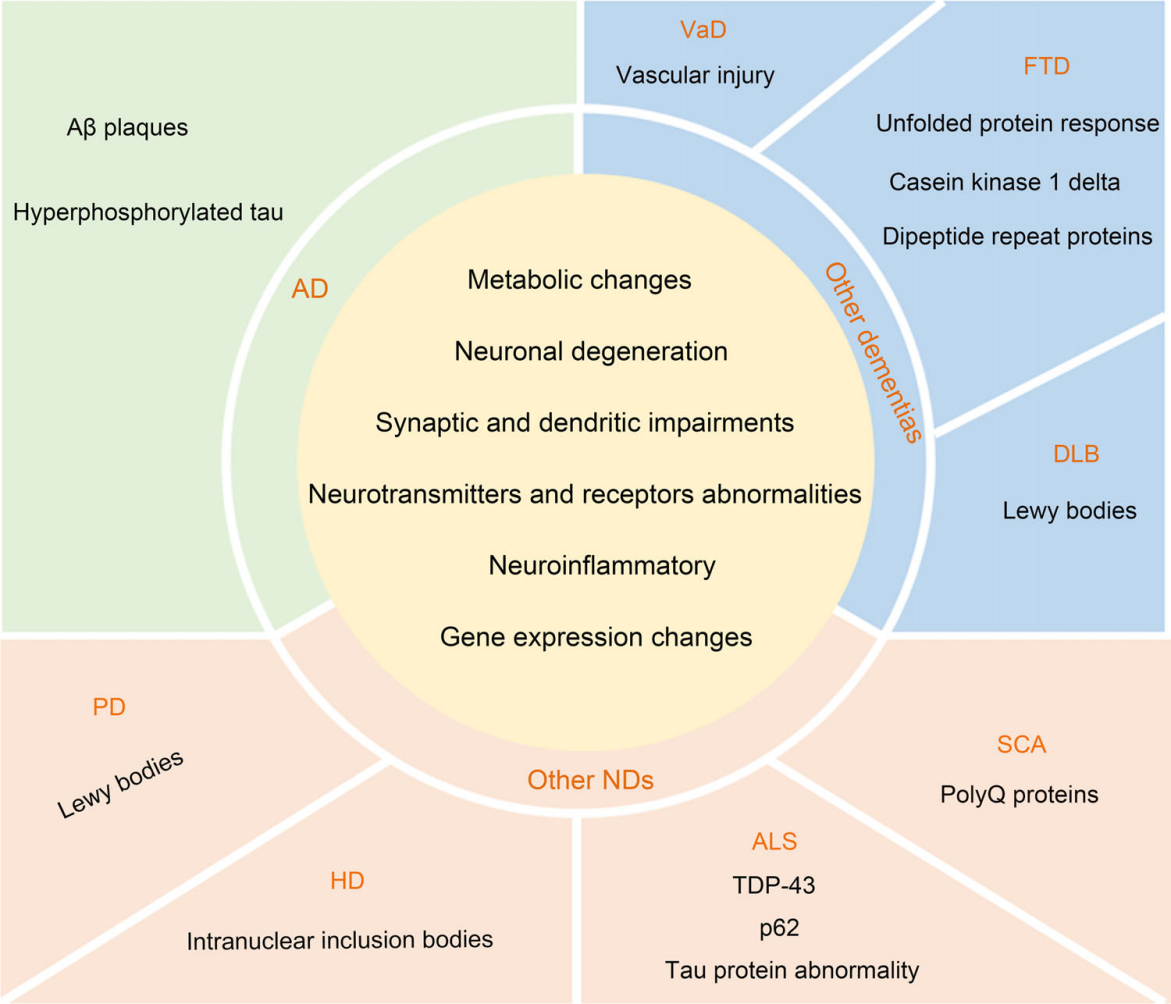
Pathological and biochemical changes of the cerebellum in AD, VaD, DLB, FTD, PD, ALS, HD, and SCA. The yellow circles highlight common pathological and biochemical changes in the cerebellum across AD, VaD, DLB, FTD, PD, ALS, HD, and SCA. The remaining sections detail disease‐specific pathological and biochemical alterations in the cerebellum. AD, Alzheimer's disease; ALS, amyotrophic lateral sclerosis; Aβ, amyloid‐β; DLB, Lewy body dementia; FTD, frontotemporal dementia; HD, Huntington's disease; PD, Parkinson's disease; polyQ, polyglutamine; SCA, spinocerebellar ataxias; VaD, vascular dementia.

PD is characterized by the loss of dopaminergic neurons in the substantia nigra.^245^ PD patients have been shown to have lower levels of cerebellar dopaminergic transmission markers compared with healthy individuals.^246^ Additionally, α‐synuclein is a key biomarker for PD based on its central role in PD pathophysiology of the disease.^247^ A study has revealed that numerous α‐synuclein‐positive, round inclusions were found in the cerebellar white matter in all the patients with PD.^216^ Furthermore, the cerebellar of all PD patients have displayed neuronal and oligodendroglial α‐synuclein aggregation pathology, with aggregation also observed in the deep cerebellar nucleus, adjacent white matter tracts, and slightly involved lobules.^248^ In addition, the degree of PC activation has been negatively correlated with the level of dopaminergic neuron loss.^249^, ^250^ Khadrawy et al.^251^ also found significant necrosis of PCs in the PD cerebellum. Furthermore, the PD cerebellum has been associated with neuroinflammation,^252^ oxidative stress,^253^ mitochondrial abnormalities,^254^ metabolic changes,^255^ and alterations in neurotransmitters and receptors.^256^ Thus, the cerebellum in PD exhibits various pathological changes (Figure 3).

Cerebellar neurodegeneration and atrophy have been well documented in PD.^257^, ^258^ Studies have found cerebellar atrophy in PD patients, particularly with greater volume loss in the grey matter of cerebellar subregions compared with controls.^258^, ^259^, ^260^ Functional imaging studies have also provided evidence that the PD cerebellum is involved in disease progression.^259^

Numerous studies have consistently demonstrated cerebellar involvement in PD. Nevertheless, the precise mechanisms underlying cerebellar dysfunction and its role in PD pathophysiology remain elusive, warranting further investigation. Elucidating the cerebellar alterations in PD could provide valuable insights into the disease pathogenesis. In light of the current clinical and basic research findings, the cerebellum should be regarded as an integral component in the study of PD.

### 4.2 Cerebellum and ALS

In addition to PD, ALS is another ND characterized by the progressive loss of motor neurons in the brain and spinal cord, ultimately leading to fatality.^261^ Traditionally, ALS has been regarded as a pure motor disorder. However, recent evidence suggests that a substantial proportion of ALS patients exhibit nonmotor symptoms (Table 1).^262^

Numerous studies have demonstrated significant structural and functional abnormalities in the primary motor cortex in ALS patients. In comparison, cerebellar abnormalities in ALS have received considerably less attention. Nevertheless, neuropathological, biochemical, and imaging studies have revealed cerebellar alterations in ALS, suggesting their potential involvement in disease progression.

ALS is characterized by evident degenerative changes in the cerebellum and spinocerebellar tract.^263^, ^264^, ^265^ In ALS patients, over 95% of affected spinal motor neurons exhibit impaired metabolism and cytoplasmic aggregates of trans‐activating response DNA‐binding protein 43 (TDP‐43).^266^ Moreover, TDP‐43 pathology has been observed in both neurons and glia of the cerebellum in ALS patients.^267^ p62, another hallmark protein in ALS, consistently appears in cytoplasmic protein inclusions within spinal cord motor neurons of sporadic ALS (sALS) cases.^268^ Studies have also revealed ubiquitinated p62‐positive inclusions in the cerebellum of ALS patients, especially in the granular layer.^269^, ^270^, ^271^ In addition to TDP‐43 and p62, alterations in the metabolism of microtubule‐associated tau protein are also evident in ALS.^272^ Abnormal tau protein pathology has been also present in the cerebellum of ALS.^273^, ^274^ Furthermore, neuroinflammation is a main hallmark of ALS.^275^ Abnormal neuroinflammation has been observed in the cerebellum of transgenic ALS mouse models.^276^, ^277^ Motor neuron degeneration is a fundamental pathological feature of ALS.^278^ Cerebellar neurons also degenerate in ALS.^276^, ^279^, ^280^, ^281^ Moreover, abnormally clear dendritic balloons are observed in the molecular and PC layers of the cerebellar anterior horn and spinal cord in ALS patients.^282^ Additionally, ultrastructural abnormalities were identified in the cerebellum of late‐stage Wobbler mice.^283^ The cerebellum in ALS also has exhibited oxidative stress abnormalities,^284^ mitochondrial damage,^285^ neurotransmitters and receptors changes,^277^ and gene changes (Figure 3).^286^, ^287^

Structural and functional imaging studies have revealed structural and functional alterations in the cerebellum of ALS patients.^288^ Several studies have demonstrated cerebellar atrophy in ALS,^257^, ^289^, ^290^ affecting both cerebellar grey matter,^291^, ^292^, ^293^, ^294^ and cerebellar white matter.^295^ Conversely, one study has reported increased white matter intensity in the cerebellum of ALS patients.^296^ In an ALS mouse model, enlarged ventricles and hypointense striations have been observed in the cerebellum.^297^ Moreover, several studies have demonstrated increased FC in the inner cerebellum and other cerebellar regions in ALS patients.^298^, ^299^, ^300^, ^301^ In contrast, some studies have reported decreased FC in the left and right cerebellar lobule VI in ALS participants.^302^, ^303^ Additionally, studies have investigated metabolic levels in the cerebellum of ALS patients, although the findings remain inconsistent.^304^, ^305^, ^306^, ^307^

### 4.3 Cerebellum and HD

HD is caused by an abnormal CAG repeat expansion in the huntingtin (HTT) gene, leading to a toxic gain of function in the mutant HTT protein.^308^ Based on the age of onset, HD is classified into three subtypes: juvenile HD,^309^ adult‐onset HD, and late‐onset HD.^310^ In HD patients, the primary sites of pathology include the caudate and lentiform nuclei, followed by the cerebral cortex (predominantly in the frontal lobe), medial globus pallidus, thalamus, and hypothalamus. HD is characterized by both motor and nonmotor symptoms.^311^

A growing body of evidence suggests that HD patients and animal models exhibit abnormal pathological and biochemical changes in the cerebellum. The cerebellum plays a dual role in HD, serving as both a protective mechanism and a disease target. Changes in the number, structure, activity, and biochemistry of cerebellar cells have been observed in both HD patients and animal models.^312^, ^313^, ^314^, ^315^, ^316^, ^317^ Moreover, postmortem examination of HD patient brains has revealed diffuse nuclear HTT immunoreactivity and intranuclear inclusion bodies.^318^ Intracellular inclusion bodies have also been identified in the cerebellum of HD patients.^319^, ^320^ Similarly, a high prevalence of huntingtin protein‐positive neuronal intranuclear inclusion bodies has been observed in the cerebellum of HD model mice.^317^, ^318^, ^321^ Furthermore, non‐ATG translation proteins associated with sense and antisense repeat expansions have been presented in the HD cerebellum, leading to neuronal loss, microglial activation, and apoptosis. Apart from the aforementioned pathological changes, alterations in cerebellar neurotransmitters and receptors have also been reported in HD.^322^, ^323^, ^324^ The cerebellum in HD has exhibited ubiquitin–proteasome system dysfunction,^325^, ^326^ upregulation of inflammatory factors,^327^ oxidative stress abnormalities,^328^ mitochondrial changes,^329^ metabolic changes,^330^ loss of presynaptic terminal integrity,^331^ severe cerebellar glycosphingolipid abnormalities,^332^ and hypermetabolism.^333^ The HD cerebellum has been presented with alterations in HD‐related genes.^334^, ^335^

Based on current research, the volume change of the cerebellum in HD remains inconclusive. Although some studies have reported no cerebellar atrophy in HD,^336^ others have suggested an increase in cerebellar volume.^337^, ^338^ Moreover, several studies have demonstrated that the extent of cerebellar atrophy varies across different regions and subtypes of HD.^339^, ^340^, ^341^, ^342^, ^343^, ^344^, ^345^, ^346^ Cerebellar atrophy in specific regions may lead to distinct clinical manifestations among HD patients.^347^, ^348^ Cerebellar volume changes may be associated with the number of CAG repeats in the HTT gene.^349^ In addition to the change in cerebellar structure in HD, there are also changes in cerebellar function.^350^, ^351^ The study also has shown that cerebellar direct current stimulation improved the motor score of HD.^352^

### 4.4 Cerebellum and SCA

SCA is an ND caused by dysfunction in the cerebellum and its afferent and efferent connections. The degeneration of PCs causes the primary symptoms of SCA.^353^, ^354^ Autopsies of SCA patients have shown severe loss of cerebellar PCs and dendritic anomalies, accompanied by significant astrocyte proliferation.^355^ Furthermore, it has been shown that SCA1 mouse PCs lack cytochrome‐c‐oxidase, and exhibit oxidative metabolism defects.^356^ Alterations in cerebellar neurotransmitters and receptors, aggregation of polyQ proteins have also been reported in SCA.^357^, ^358^, ^359^ Multiple metabolites are altered in the cerebellum of SCA and contribute to disease diagnosis.^360^, ^361^ Meanwhile, numerous studies have demonstrated a significant reduction in cerebellar volume in patients with SCA1, 2, 3, 28, and 36.^362^, ^363^, ^364^ Moreover, several studies have demonstrated that the extent of cerebellar atrophy varies across different regions and subtypes of SCA.^365^, ^366^ Cerebellar atrophy in specific regions may lead to distinct clinical manifestations among SCA patients.^362^, ^367^, ^368^, ^369^

## 5 CONCLUSION AND PROSPECTS

The cerebellum, that stripey “little brain,” contains more than half of the neurons in the entire nervous system.^370^ The cerebellum has traditionally been considered primarily a motor structure.^370^ Does the cerebellum influence nonmotor behavior? Indeed, a wealth of evidence from clinical, neuroanatomical, neuroimaging, and electrophysiological studies has substantially shown that the cerebellum is also involved in regulating nonmotor behaviors such as attention, executive control, language, working memory, learning, pain, mood, and addiction.^9^ The functional diversity of the cerebellum is largely believed to be derived from its extensive connections and its mostly invariant architecture.^371^

The cerebellum has largely been overlooked in mainstream AD research, indicating that the current research approach largely neglects the cerebellum's potential role in AD. The exploration into the cerebellum's involvement in AD is notably nascent, especially when juxtaposed with the extensive body of research dedicated to the cortex and hippocampus's roles in AD. This scarcity of cerebellum‐focused studies might lead to the premature conclusion that the cerebellum's contributions to cognitive and affective behaviors are minimal. Yet, ongoing research consistently implicates the cerebellum in brain development and suggests that it plays a significant role in regulating functions affected by AD pathology. Despite the absence of direct evidence linking cerebellar damage to AD's clinical manifestations, the cerebellum is emerging as a critical area of interest in AD research. This interest is fueled by an increasing number of studies identifying pathological and biochemical profiles, imaging alteration, and electrophysiological changes in the cerebellum in AD, which could offer new perspectives on the disease's pathogenesis and progression. The cerebellum's structure is relatively uniform, consisting of 10 subdivisions, three cortical layers, and one white matter layer. Therefore, further research is needed to clarify how these pathological and biochemical profiles, imaging alterations, and electrophysiological changes are distributed across different regions and layers of the cerebellum throughout AD. Questions regarding the regional or layer‐specific patterns of Aβ deposition in the cerebellum, and how these patterns might illuminate the progression of AD or correlate with clinical symptoms, are particularly intriguing. Exploring whether the spatial distribution of these changes is associated with cortical and hippocampal lesions or aligns with specific clinical manifestations could significantly enhance our comprehension of AD and its impact on the cerebellum.

It is worth noting that in certain investigations, there are no obvious abnormalities found within the cerebellum, even in the disease's advanced phases.^372^ This could be attributed to what is known as the cerebellar reserve, a concept that describes the cerebellum's capacity to compensate for and recuperate from injuries.^373^ This distinctive capability of the cerebellum is credited to various forms of synaptic plasticity, encompassing both multimodal and redundant inputs to the cerebellum—two principal characteristics of cerebellar circuitry.

The cerebellum's role in the progression of AD remains enigmatic, whether it serves as the disease's genesis, an early vulnerability zone, or a protective barrier. Additional research is urgently needed to clarify this aspect. Currently, the clinical evidence regarding the cerebellum in AD is limited to small sample sizes and even individual case reports. Animal studies exploring the cerebellum in AD are still in their early observation stages, and explicit molecular pathways remain unexplored. Consequently, the study of the cerebellum in AD is still in its infancy, and ongoing research is imperative to unravel its function in this disease. Although the cerebellum has been more extensively studied in PD compared with AD, the aforementioned challenges persist. Furthermore, ongoing investigations of the cerebellum in VaD, DLB, FTD, ALS, HD, and SCA continue to hint at its potential significance in neurologic disorders.

**FIGURE 4:**
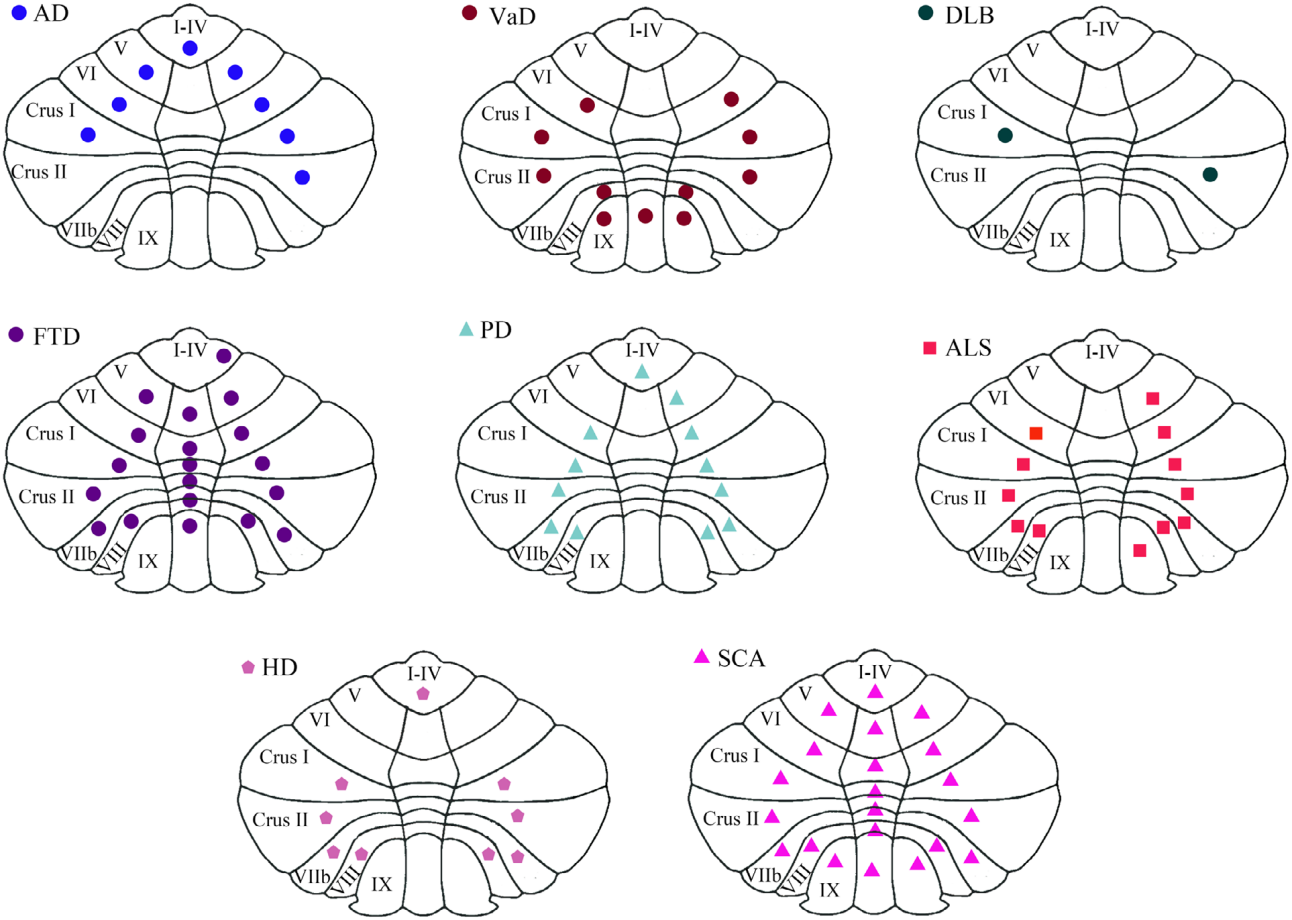
Atrophic distribution of cerebellum in AD, VaD, DLB, FTD, PD, ALS, HD, and SCA. AD, Alzheimer's disease; ALS, amyotrophic lateral sclerosis; DLB, Lewy body dementia; FTD, frontotemporal dementia; HD, Huntington's disease; PD, Parkinson's disease; SCA, spinocerebellar ataxias; VaD, vascular dementia.

AD, other dementias, and other NDs all show different clinical manifestations‐ (Table 1), and corresponding pathological and biochemical alterations, and imaging changes are also found in the cerebellum (Figures 3 and 4).

AD, other dementias, and other NDs have similar pathological and biochemical changes, including neuronal degeneration, synaptic and dendritic dysfunctions, neuroinflammatory changes, neurotransmitters and receptors abnormalities, metabolic changes, and gene changes. Compared with other dementias and NDs, there are Aβ plaques and p‐Tau in the cerebellum of AD. Combined with the different clinical manifestations of AD, this suggests that Aβ plaques and p‐Tau may have characteristic damage patterns in the cerebellum. In addition, the atrophy site of AD cerebellum is different from that of other NDs, but whether these differences have the characteristics of the disease still needs to be further explored. If the cerebellar atrophy distribution is characteristic in different diseases, then the cerebellar atrophy distribution may be used as a differentiating point between different diseases.

Overall, the cerebellum remains understudied in AD, other dementias, and other NDs. One of the major reasons is that the cerebellum has been neglected in the study of AD, other dementias, and other NDs. We are still far from fully understanding the role of the cerebellum and what happens if the cerebellum does not work properly. In addition, with only five main types of neurons in the cerebellum, one wonders how information from the spinal cord and brainstem to the regions such as the hypothalamus and basal ganglia converge in the cerebellum in a coherent circuit. Our interest in the role of the cerebellum in AD, other dementias, and NDs is increasing. In this regard, it is crucial to explore the mechanisms underlying the cerebellum's involvement in the pathogenesis of AD, other dementias, and other NDs from both fundamental and clinical perspectives.

## AUTHOR CONTRIBUTIONS

Cui Yang wrote the paper and made the figures and tables. Guangdong Liu and Xi Chen prepared the article. Weidong Le initiated the concept, helped with the revision of the manuscript, and provided supervision of the project and funding acquisition. Xi Chen contributed to the project administration, review, and editing. All authors have read and approved the final manuscript.

## CONFLICT OF INTEREST STATEMENT

The authors declare no conflict of interest.

## ETHICS STATEMENT

Not applicable.

## Data Availability

Not applicable.
